# Association between helicobacter pylori infection and primary open-angle glaucoma: a systematic review and meta-analysis

**DOI:** 10.1186/s12886-023-03111-z

**Published:** 2023-09-11

**Authors:** Elnaz Ezzati Amini, Yousef Moradi

**Affiliations:** Social Determinants of Health Research Center, Research Institute for Health Development, Kurdistan University of Medical Sciences, Sanandaj, Iran

**Keywords:** Helicobacter Pylori, Glaucoma, Primary Open-Angle Glaucoma, Pseudo-exfoliation Glaucoma, Normal tension Glaucoma, Ocular hypertension

## Abstract

**Purpose:**

This systematic review and meta-analysis summarize the evidence for the association between Helicobacter pylori infection and Primary Open-Angle Glaucoma.

**Methods:**

Eligible studies reporting an association between H. pylori infection and Glaucoma were identified through an extensive search of the Excerpta Medica (EMBASE), Web of Science, Scopus, and PubMed databases and an assessment of the reference list of the top articles until October 2022. Analysis was performed with random effects model using Stata 16.

**Result:**

Twenty-four studies were included in the systematic review. This study involved 1602 glaucoma patients and 2800 control individuals. The combined RRs of cohort studies and overall combined ORs of case-control studies showed a significant correlation between H. pylori infection and Glaucoma. Subgroup analysis showed that glaucoma patients had a higher risk of having H. pylori infection if they were residents of Europe countries (Cohort: RR: 1.69; 95% CI: 1.3–2.19) and (Case-Control: RR: 3.71; 95% CI: 2.07–6.64), if they had POAG type (Cohort: RR: 1.76; 95% CI: 1.37–2.27) and (Case-Control: RR: 3.71; 95% CI: 2.934.70), if their diagnostic method of HP was histology (Cohort: RR: 1.95; 95% CI: 1.26–3.01) and (Case-Control: RR: 4.06; 95% CI: 2.28–7.22), and if they were over 60 years old (Cohort: RR: 1.63; 95% CI: 1.33-2.00) and (Case-Control: RR: 2.95; 95% CI: 2.27–3.83).

**Discussion:**

The results of this meta-analysis suggest a statistically significant association between Helicobacter pylori infection and Primary Open-Angle Glaucoma.

## Introduction

Helicobacter pylori (HP) is a spiral-shaped and gram-negative Micro-organism that affects the epithelial mucosa of the stomach [1, 2]. It is estimated that approximately 50% of the world’s population is infected with H. pylori [3]. HP is the principal cause of peptic ulcer disease, chronic gastritis, and gastric cancer [1, 2]. In addition to these common diseases, extra-gastrointestinal manifestations of H. pylori have recently attracted the interest of many researchers [2], including blood diseases such as iron deficiency anemia, vitamin B12 deficiency, and chronic immune thrombocytopenia, metabolic syndrome, diabetes, non-alcoholic fatty liver disease, Alzheimer’s disease, neurologic disease, skin disease, cardiovascular disease, and eye disease [3–7]. Ocular manifestations of H. pylori infection include glaucoma, central serous chorioretinopathy, blepharitis, and uveitis [2].

**Fig. 1:**
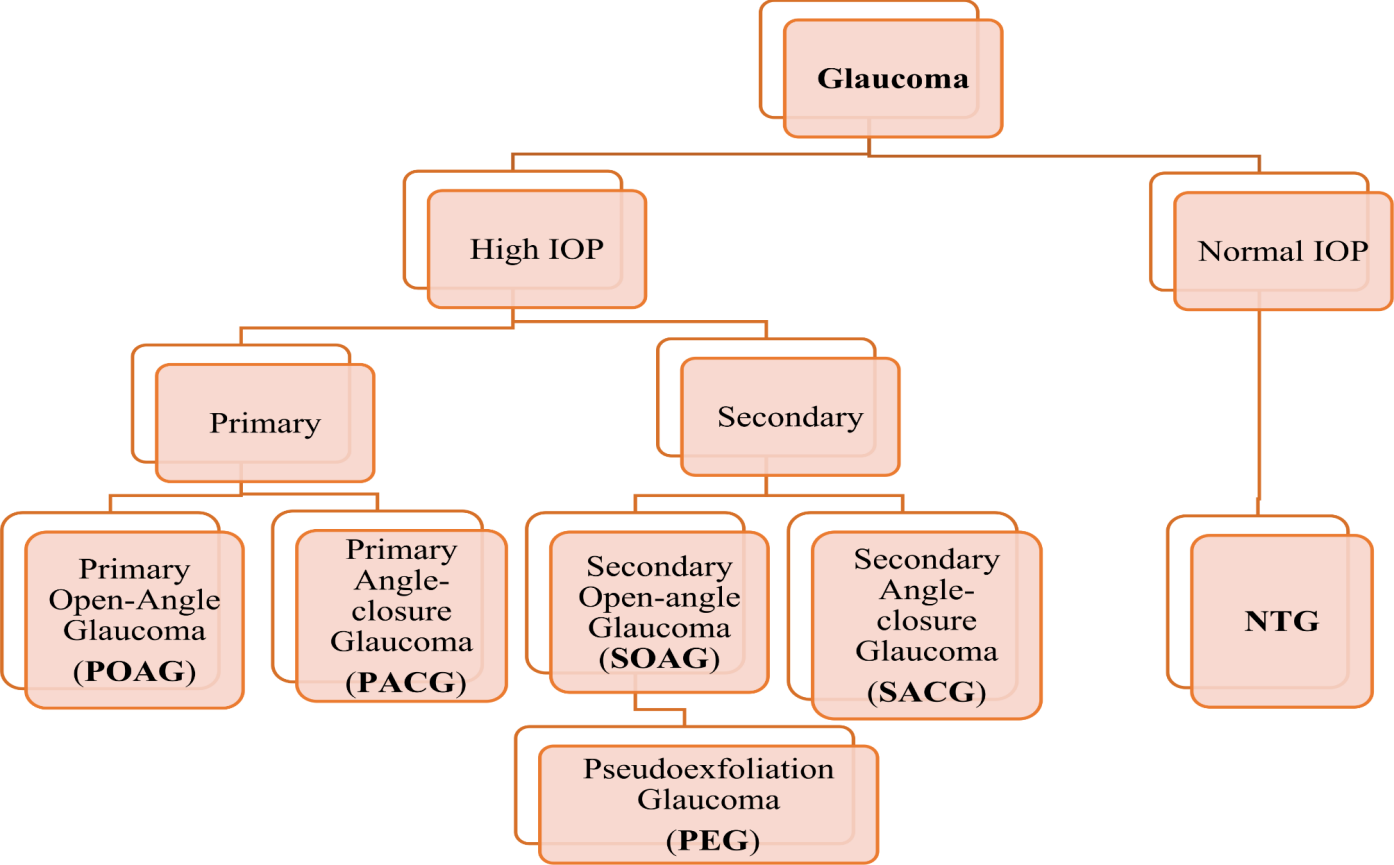
Glaucoma Types [9]

Glaucoma is the acquired loss of retinal ganglion cells (RCG) and axons within the optic nerve or optic neuropathy [8]. Glaucomatous neuropathy is mainly caused by persistent elevated intraocular pressure (IOP above 22 mmHg). However, if the pressure is within the normal range, it can develop normal tension glaucoma(NTG), which is infrequent (Fig. 1) [9, 10]. The two main types are open-angle glaucoma and closed-angle glaucoma [11], and primary open-angle glaucoma (POAG) is the most typical form in the United States [12]. Glaucoma is the prominent cause of blindness globally and occurs most commonly in the elderly [13–15]. It is estimated to affect about 76 million of the world’s population between the ages of 40 and 80. By 2040, this number is expected to increase to 111.8 million worldwide [14, 16, 17].

The hypothesis of the correlation between H. pylori infection and glaucoma originated from the role of H. pylori causing the release of various pro-inflammatory and vasoactive substances, arteriosclerosis-induced increased platelet activation and aggregation, and impact on the trabecular meshwork cell apoptotic process. Cross-reactivity between antibodies to Helicobacter pylori and the ciliary epithelium has also been speculated [18–22].

As studies evaluating potential associations between H. pylori infection and POAG have reported positive [19, 20, 23–38] or no association [39–44], Controversy still exists. For this reason, this study aimed to elucidate the putative link between primary open-angle glaucoma (POAG) and H. pylori infection in all possible combinations. Glaucoma guidelines need to be updated, so the results of this study can be helpful for this goal.

## Methods, search terms, and search strategies

This study aimed to address the Association between Helicobacter pylori Infection and POAG through systematic review and meta-analysis based on the PRISMA guidelines for systematic reviews and meta-analysis of cohort and case studies [45]. A systematic electronic literature search, a full search of articles through October 2022, was performed in the online databases of PubMed (Medline), Embase, Scopus, and Web of Science. The investigation was conducted based on keywords selected by Mesh and Emtree. The first ten pages of Google Scholar were also examined to prevent the loss of gray articles according to the keywords. We also discussed these studies’ references for possible relevant articles (Manual Search). The keywords searched on the international site included “helicobacter pylori” with its synonyms (“Campylobacter Pylori,” “Campylobacter Pyloridis,” “Campylobacter Pyloris,” “Helicobacter Nemestrinae”) and “Glaucoma” with its synonym (“Primary Open-angle Glaucoma,” “POAG,” “Chronic Glaucoma”).

### Eligibility criteria

In the current meta-analysis, the following inclusion and exclusion criteria were required to be met. The inclusion criteria were: cohorts or case-control studies evaluating the association between H. pylori infection and Glaucoma and their results using the indicators measuring the association, such as OR, RR, and HR with a 95% confidence interval. The exclusion criteria were duplicate data, Cross-sectional studies, editorials, letters, review articles, case reports, systematic reviews, and meta-analyses, intervention studies (RCT), and articles not reporting the outcomes of interest.

### Selection and screening

At the end of the search, all screened studies were uploaded to Endnote version 9. After identifying and removing duplicates, studies still needed to be published and were in the peer-reviewed phase were excluded. After that, the full texts of the screened articles were reviewed, and the ones which met the desired criteria were selected for meta-analysis. If multiple types of glaucoma were reported in an article, each kind combined with the control group was extracted as an independent dataset.

### Data extraction and quality assessment

After selecting the studies, the required data were extracted by the two independent researchers (EEA and YM) and recorded in Excel. After examining the discrepancies and reaching a single result, the OR and RR reported with a 95% confidence interval and related to the association between H. pylori and POAG were extracted from the studies. Then, this information was removed, including the first author’s name, year of publication, Type of study, country of origin, Host Journal, sample size, Glaucoma Cases, Controls, Mean Age (Years), Glaucoma Subtype, H. pylori Infection Diagnosis, NOS Score and Relationship between H. pylori and Glaucoma (OR, CI).

The quality of the studies in this meta-analysis was assessed using the Newcastle Ottawa Scale (NOS) [46, 47]. On this scale, a maximum of 9 points can be awarded to each study in three sections: selection of participants (4 points), comparison (2 points), and results (3 points). Studies are divided into ones with high quality (7–9 points), medium quality and high risk of bias (4–6 points), and low quality and high risk of bias (0–3 points).

### Statistical analysis

In this systematic review, seven cohort and seventeen case-control studies were included in the analysis. The researchers have decided to perform the final analysis on both cohort and case-control studies since they are methodologically different, and their indicators of measuring the association are various. Analyzes related to these studies were performed by Stata software version 16. First, the logarithm and the standard deviation (SD) of the relative risk (RR) logarithm were calculated using a combination of seven cohort studies. Second, the logarithm and the standard deviation of the logarithm of Odd’s Ratio (OR) were computed using a combination of seventeen case-control studies. The model of fixed effects was used to estimate the pooled risk ratio. Also, subgroup analyses were performed to identify the primary source of heterogeneity by considering the Continents where the study was conducted, the type of Glaucoma, the Diagnostic Method of HP, and the participants’ age. I square index and Cochrane Q test were used to determine heterogeneity. Egger and funnel plot tests were used to evaluate the publication bias. The significance level was considered below 0.05 in this study.

## Results

### Qualitative results

After completing the search in international databases, 826 articles were retrieved. First, duplicates (541) were removed, then 285 articles were entered into the screening stage according to the title. One hundred sixty-six articles were removed at this step, and 119 papers were evaluated based on their abstracts, considering the inclusion and exclusion criteria. Then, forty-eight pieces remained and entered the screening phase based on their full texts. Of these articles, twenty studies were excluded due to the non-related outcomes to the present study and eight cases because of different statistical populations. Finally, twenty studies related to the subject and purpose of the research had the necessary conditions to be entered into the study. After a manual search, four articles were added to the final studies [33, 36, 37, 48].

**Fig. 2:**
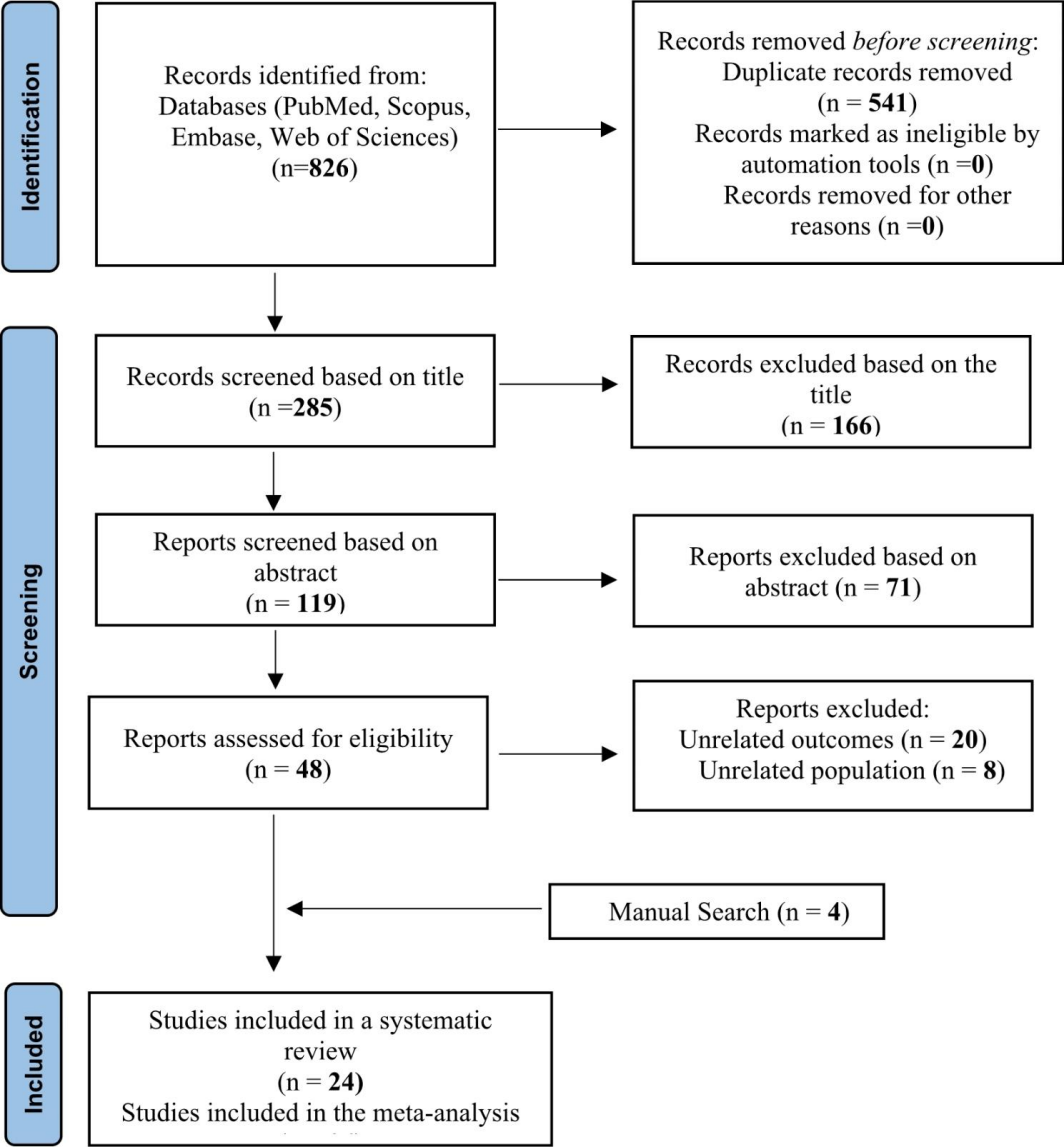
Flowchart based on Preferred Reporting Items for Systematic Reviews and Meta-Analyses (PRISMA) guidelines interpreting the selection process of the reviewed studies to insert in the meta-analysis

After screening, twenty-four articles were finally selected, of which seven articles were cohorts (Tsolaki et al. [6], Kim et al. [18], Kountouras et al. [23, 24], Abrishami et al. [25], Kurtz et al. [39] and Galloway et al. [40]). Seventeen articles were case-control (Kountouras et al. [16], Jahadi et al. [38], Abd Elahi et al. [39], Hong et al. [29], Deshpande et al. [27], Zavos et al. [24], Peng et al. [30], Samarai et al. [25], Shasha et al. [31], Tuzcu et al. [40], Noche et al. [41], Zhang et al. [34], Xinjifu et al. [33], Sultana et al. [26], Raji et al. [32], Zhou et al. [35], and Alkaffas et al. [46]) which were entered meta-analysis (Fig. 2). A diagram of how to select the final studies is shown in Fig. 2. Table 1 lists the general characteristics of the included studies.

**Table 1:** The characteristics of Studies Included in this Meta-Analysis

No.	First Author	Year of Publication	Type of Study	Country (Region)	Host Journal	Glaucoma Cases	Controls	Mean Age (Years)*	Controls Deposit	Glaucoma Subtype	H. pylori Infection Diagnosis	NOS Score	Relationship between H. pylori and Glaucoma (OR, CI)
**1**	Kountouras et al [19]	2001	Case-Control	Greece	Ophthalmology	9	30	62	Anemia	PEG	HISTOLOGY	6	9.14 (1.01–82.44)
**2**	Kountouras et al [19]	2001	Case-Control	Greece	Ophthalmology	32	30	64	Anemia	POAG	HISTOLOGY		8 (2.25–28.48)
**3**	Kountouras et al [23]	2002	Cohort	Greece	Arch Intern Med	41	30	61.4	Anemia	POAG	HISTOLOGY	6	8.23 (2.53–26.75)
**4**	Kountouras et al [24]	2003	Cohort	Greece	Graefe’s Arch Clin Exp Ophthalmol	26	31	69.2	Cataract	POAG	ELISA	6	25 (1.38-452.34)
**5**	Kountouras et al [24]	2003	Cohort	Greece	Graefe’s Arch Clin Exp Ophthalmol	27	31	70.6	Cataract	PEG	ELISA		2.35 (0.63–8.76)
**6**	Jahadi et al [41]	2004	Case-Control	Iran	Journal of Medical Research	60	30	60.2	Healthy	POAG	ELISA(Serum)	7	2.43 (0.85,6.97)
**7**	Jahadi et al [41]	2004	Case-Control	Iran	Journal of Medical Research	60	35	60.2	Healthy	POAG	ELISA(Stool)		1.42 (0.35,5.68)
**8**	Galloway et al. [40]	2003	Cohort	Canada	Ophthalmology	38	94	63.2	Participants without glaucoma	POAG	ELISA	8	1.41 (0.59–3.4)
**9**	Galloway et al. [40]	2003	Cohort	Canada	Ophthalmology	16	94	73.2	Participants without glaucoma	PEG	ELISA		1.32 (0.38–4.54)
**10**	Galloway et al. [40]	2003	Cohort	Canada	Ophthalmology	19	94	67.7	Participants without glaucoma	NTG	ELISA		1.41 (0.45–4.4)
**11**	Galloway et al. [40]	2003	Cohort	Canada	Ophthalmology	24	94	62.6	Participants without glaucoma	OHT	ELISA		1.24 (0.55,2.79)
**12**	Abd elahi et al [42]	2005	Case-Control	Iran	Journal of current Ophthalmology	34	34	60	Cataract	POAG	ELISA	7	1.94 (0.70,5.41)
**13**	Abrishami et al. [25]	2007	Cohort	Iran	Bina J Ophthalmol	44	79	60.8	Cataract	POAG	ELISA	7	3.69 (1.68–8.13)
**14**	Hong et al. [32]	2007	Case-Control	China	Asian J Ophtholmol	24	24	63.9	Participants without glaucoma	POAG	UBT	8	4.49 (1.26-16)
**15**	Deshpande et al. [30]	2008	Case-Control	India	J Glaucoma	50	50	63.7	Cataract	POAG	ELISA	8	1.20 (0.52–2.79)
**16**	Deshpande et al. [30]	2008	Case-Control	India	J Glaucoma	50	50	67	Cataract	PEG	ELISA		0.41 (0.18–0.91)
**17**	Kurtz et al. [39]	2008	Cohort	Israel	J Glaucoma	13	36	67.7	Cataract	POAG	ELISA	8	0.74 (0.21–2.67)
**18**	Kurtz et al. [39]	2008	Cohort	Israel	J Glaucoma	23	36	67.7	Cataract	PEG	ELISA		1.19 (0.4–3.54)
**19**	Kurtz et al. [39]	2008	Cohort	Israel	J Glaucoma	15	36	67.7	Cataract	NTG	ELISA		0.96 (0.28–3.27)
**20**	Kim et al. [18]	2011	Retrospective Cohort	South Korea	IOVS	100	88	55.6	Healthy	NTG	ELISA	8	2.05 (1.12–3.75)
**21**	Kim et al. [18]	2011	Retrospective Cohort	South Korea	IOVS	104	1116	53.4	Healthy	NTG	ELISA		1.83 (1.17–2.86)
**22**	Zavos et al. [27]	2012	Case-Control	Greece	Ophthalmic Res	51	35	71.4	Anemia	POAG	HISTOLOGY	8	5.69 (2.08–15.54)
**23**	Peng et al [33]	2012	Case-Control	China	J Bethune Military. Medical College	37	34	42	Healthy	POAG	ELISA	7	5.86 (2.06,16.64)
**24**	Samarai et al. [28]	2014	Case-Control	Iran	Global Journal of Health Science	37	42	73.05	Cataract	POAG	ELISA	8	5.61 (1.68–18.75)
**25**	Shasha et al. [34]	2014	Case-Control	China	Master’s Thesis	30	30	61.86	Cataract	POAG	UBT	7	8.50 (2.37,30.47)
**26**	Shasha et al. [34]	2014	Case-Control	China	Master’s Thesis	30	30	64.2	Cataract	PACG	UBT		1.49 (0.54,4.14)
**27**	Tsolaki et al. [6]	2015	Cohort	Greece	Ophthalmology	35	31	62.18	Participants without glaucoma	POAG	HISTOLOGY	7	2.65 (0.97–7.24)
**28**	Tuzcu et al. [43]	2015	Case-Control	Turkey	Arq Bras Oftalmol	35	35	59.08	Participants without glaucoma	POAG	UBT	8	1.41 (0.55–3.62)
**29**	Noche et al. [44]	2016	Case-Control	Cameroon	Ophthalmology and Eye Diseases	50	31	58.52	Participants without glaucoma	POAG	ELISA	8	0.42 (0.12–1.43)
**30**	Zhang et al [37]	2016	Case-Control	China	Chinese Journal of Gerontology	83	50	60.3	Healthy	POAG	UBT	7	5.38 (2.51–11.53)
**31**	Zhang et al [37]	2016	Case-Control	China	Chinese Journal of Gerontology	82	50	59.7	Healthy	PACG	UBT		2.14 (1.03–4.43)
**32**	Xinjifu et al. [36]	2017	Case-Control	China	China Medical Guide	70	70	72.51	Healthy	POAG	ELISA	7	15.84 (6.51–38.54)
**33**	Sultana et al. [29]	2019	Case-Control	Bangladesh	BSMMU J BMC	40	40	51.4	Participants without glaucoma	POAG	ELISA	8	3.89 (1.53–9.87)
**34**	Sultana et al. [29]	2019	Case-Control	Bangladesh	BSMMU J BMC	40	40	51.4	Participants without glaucoma	POAG	UBT		7.00 (2.62–18.74)
**35**	Raji et al. [35]	2021	Case-Control	India	Journal of Clinical Research and Ophthalmology	50	50	52.02	Participants without glaucoma	POAG	HISTOLOGY	7	1.88 (0.76–4.69)
**36**	Zhou et al. [38]	2021	Case-Control	China	International Journal of Immunology and Microbiology	30	30	68	Cataract	POAG	DIGFA	7	5.68 (1.84–17.49)
**37**	Zhou et al. [38]	2021	Case-Control	China	International Journal of Immunology and Microbiology	30	30	68	Cataract	PACG	DIGFA		1.32 (0.47–3.72)
**38**	Alkaffas et al. [49]	2022	Case-Control	Egypt	Egyptian Journal of Medical Microbiology	63	30	57	Healthy	POAG	ELISA	7	6.18 (2.34–16.34)

* Refers to glaucoma group, CI; Confidence Interval, ELISA; Enzyme-Linked Immunosorbent Assay, H. pylori; Helicobacter pylori, MD; Differences of Means, N/A; Not Available, NOS; Newcastle-Ottawa scale, NTG; Normal Tension Glaucoma, OAG; Open Angle Glaucoma. OR; Odds Ratio, PEG; Pseudo-Exfoliation Glaucoma, UBT: Urea Breath Test, DIGFA: dot immunogold filtration assay, PACG: Primary angle closure glaucoma.

### Quantitative results

**Fig. 3:**
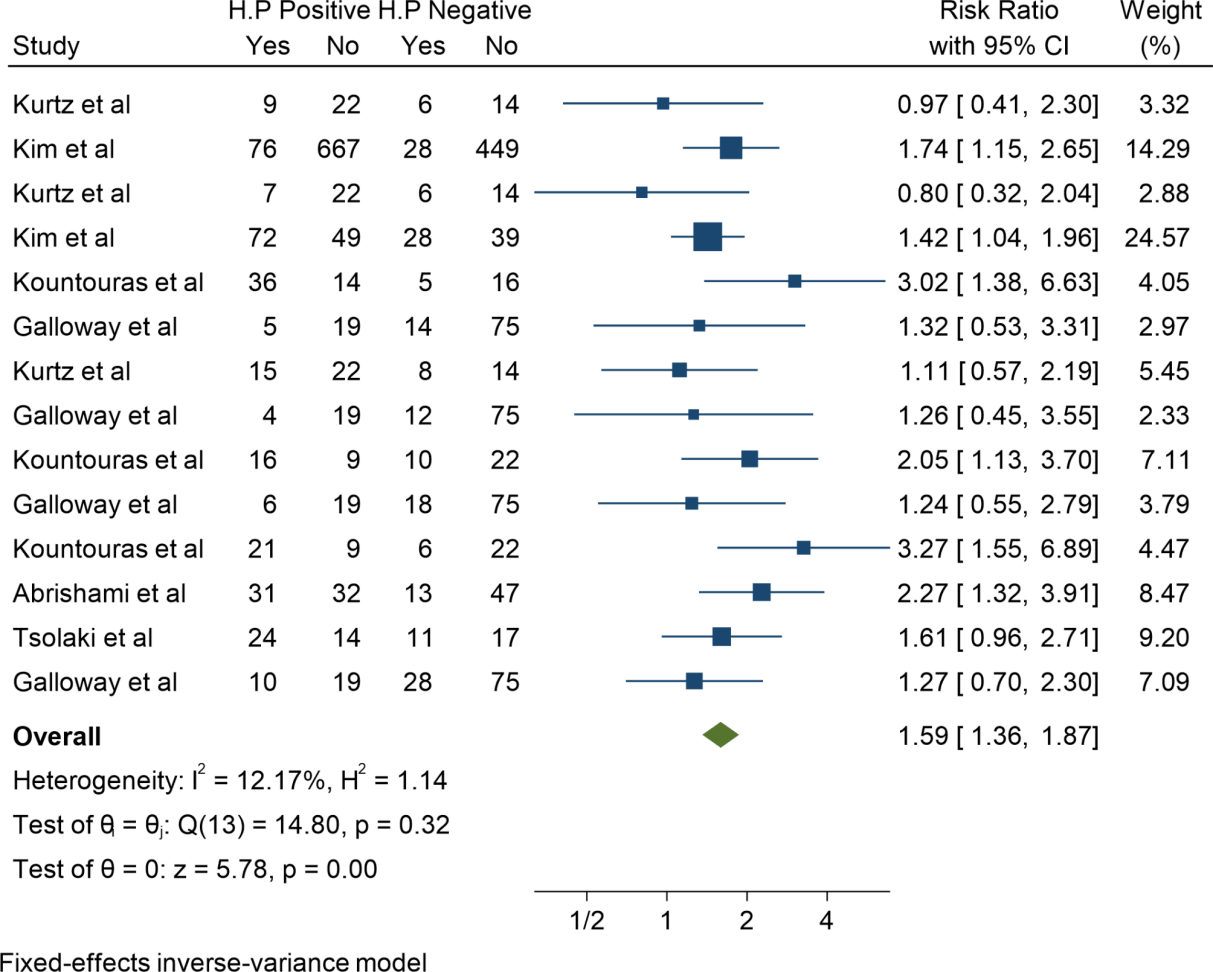
The pooled risk ratio of association between H. pylori and Glaucoma in Cohort studies

The quantitative part of the present meta-analysis has 7 cohort studies with 14 effect sizes. In these articles, the smallest reported effect size of the association between H. pylori and Glaucoma belonged to the study of Kurtz et al. with a risk ratio of 0.80 (95% CI; 0.32–2.04), and the highest effect belonged to the study of Kountouras et al. with the risk ratio of 3.27 (95% CI; 1.55–6.89). Finally, after combining these cohort studies, the pooled risk ratio of association between H. pylori and Glaucoma was 1.59 (95% CI; 1.36–1.87). The heterogeneity in this analysis was low and equal to 12.17%, with a significance level of 0.001 (Fig. 3).

**Fig. 4:**
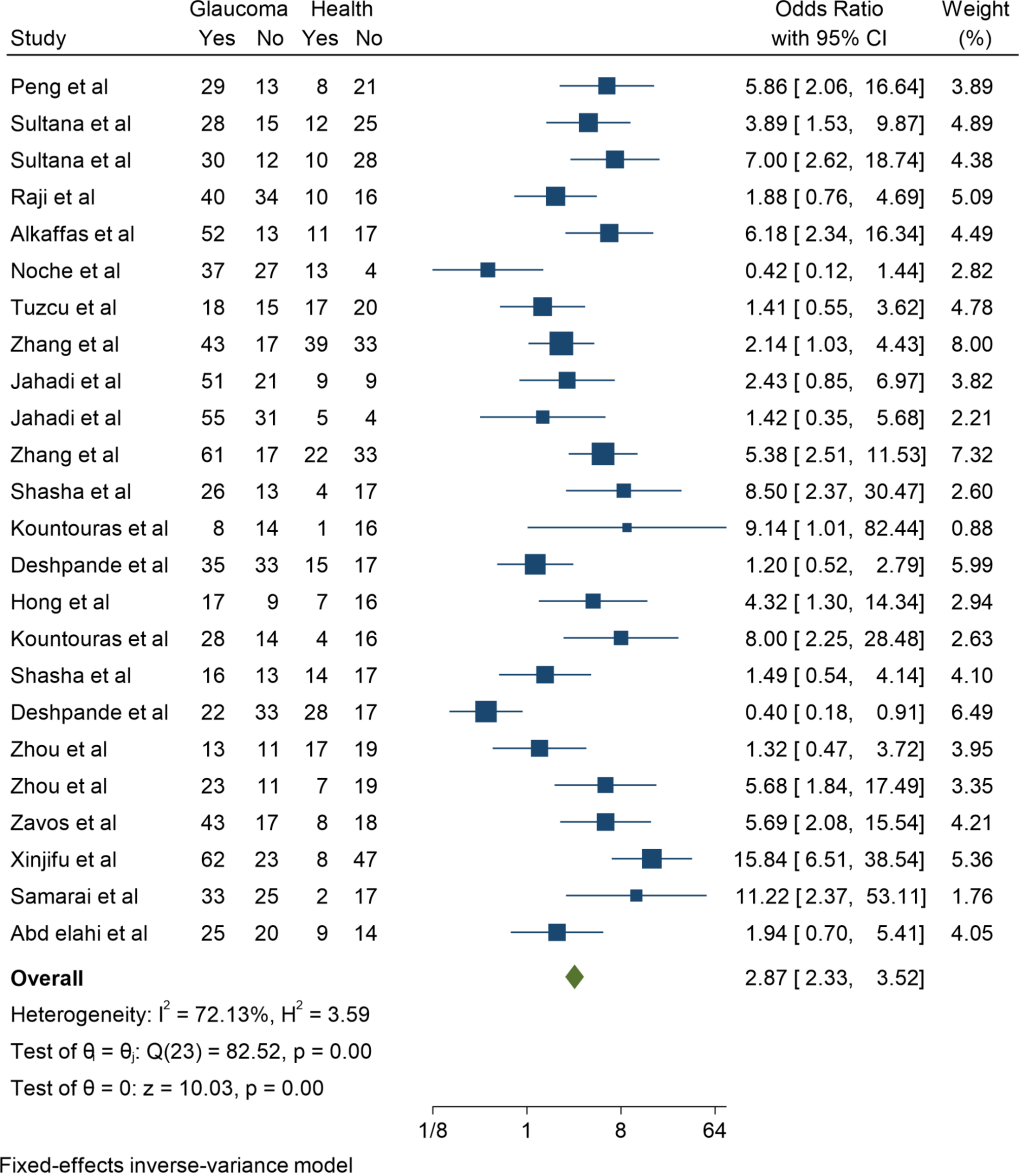
The pooled odds ratio of association between H. pylori and Glaucoma in Case-Control studies

Also, this meta-analysis has 17 case-control studies with 24 effect sizes. In these papers, the smallest reported effect size of the association between H. pylori and Glaucoma belonged to the study of Deshpande et al. with an odds ratio of 0.40 (95% CI; 0.18–0.91), and the highest effect belonged to the study of Xinjifu et al. with the odd’s ratio of 15.84 (95% CI; 6.51–38.54). Finally, after combining these case-control studies, the pooled odds ratio of association between H. pylori and Glaucoma was 2.87 (95% CI; 2.33–3.52). The heterogeneity in this analysis was equal to 72.13%, with a significance level of 0.001 (Fig. 4).

### Publication bias

**Fig. 5:**
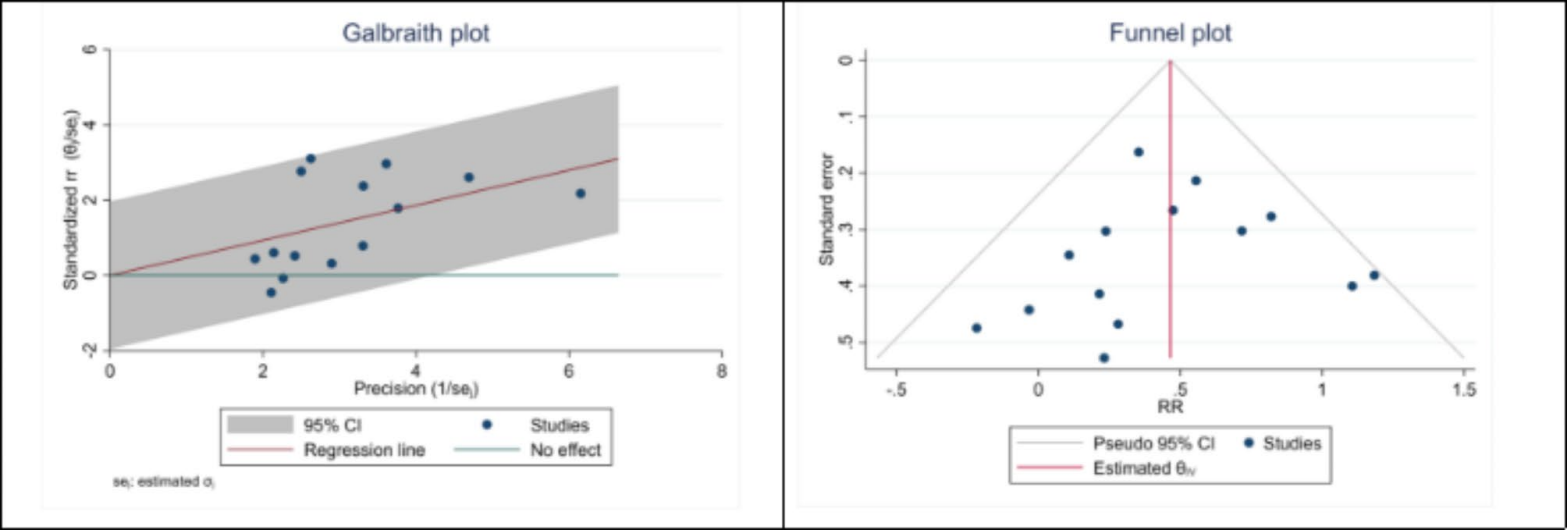
The funnel and Galbraith plot of the association between H. pylori and Glaucoma in Cohort studies

**Fig. 6:**
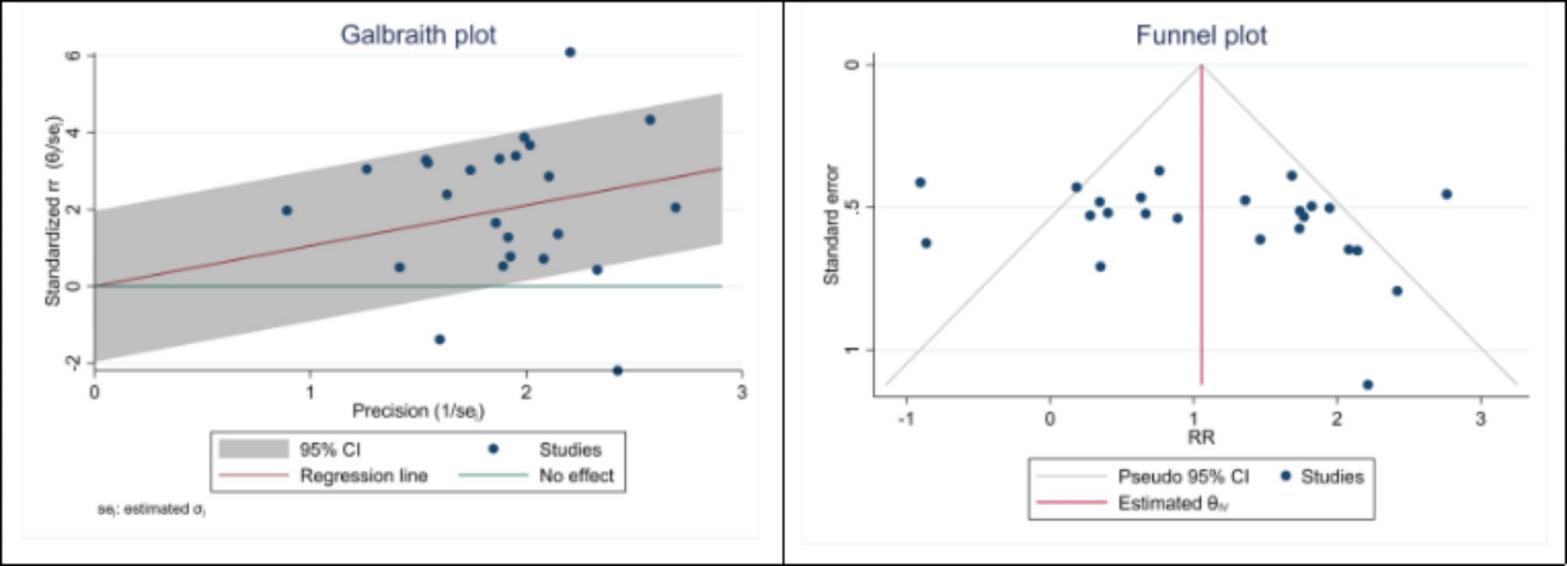
The funnel and Galbraith plot of the association between H. pylori and Glaucoma in Case-Control studies

The Egger test was used to examine and determine the publication bias according to the number of articles included in the analysis. Based on the results obtained from the Egger test, evident and significant publication bias (P < 0.001) was observed in the combination of studies included in our analysis. However, the method of cutting and filling the collected effect size did not change the results, which showed that the study results were not affected by publication bias (Figs. 5 and 6).

### Subgroup analysis

The results of subgroup analysis based on the continent where the study was conducted, Type of Glaucoma, Diagnostic Method of HP, and Age are shown in Table 2.

#### Based on the geographical location

Subgroup analysis based on the geographical location in the present meta-analysis showed that glaucoma patients had a higher risk of having H. pylori infection if they were residents of Europe countries (Cohort: RR: 1.69; 95% CI: 1.3–2.19) and (Case-Control: RR: 3.71; 95% CI: 2.07–6.64) (Table 2).

#### Based on the type of glaucoma

Subgroup analysis based on the type of Glaucoma in the present meta-analysis showed that glaucoma patients had a higher risk of having H. pylori infection if they had POAG type. (Cohort: RR: 1.76; 95% CI: 1.37–2.27) And (Case-Control: RR: 3.71; 95% CI: 2.934.70) (Table 2**).**

#### Based on the diagnostic method of HP

Subgroup analysis based on the diagnostic method of HP in the present meta-analysis showed that glaucoma patients had a higher risk of having H. pylori infection if their diagnostic method of HP was histology. (Cohort: RR: 1.95; 95% CI: 1.26–3.01) And (Case-Control: RR: 4.06; 95% CI: 2.28–7.22) (Table 2**).**

#### Based on the age of participants in the study

In this meta-analysis, the age of glaucoma patients in the selected preliminary studies was divided into groups of less than or equal to 60 years and more than 60 years. Analysis showed that glaucoma over the age of 60 years had a higher risk of having H. pylori infection (Cohort: RR: 1.63; 95% CI: 1.33-2.00) and (Case-Control: RR: 2.95; 95% CI: 2.27–3.83) (Table 2).

**Table 2:** Subgroup analysis regarding the relationship between Pylori infection and glaucoma

Study Design	Variables	Pooled Risk Ratio	% 95 Confidence Interval	Heterogeneity assessment between studies		Heterogeneity assessment between subgroup	
				I square	P value	Q test	P value
**Cohort**	**Continents** Asia Europe America	1.65 1.69 1.27	1.31–2.07 1.30–2.19 0.86–1.88	9.88% 45.62% 0.00%	0.33 0.09 1.00	14.80	0.32
	**Type of Glaucoma** NTG OHT PEG POAG	1.47 1.24 1.69 1.76	1.16–1.86 0.55–2.79 1.08–2.65 1.37–2.27	0.00% 0.00% 57.90% 27.95%	0.66 0.89 0.09 0.23	1.49	0.68
	**Diagnostic Method of HP** ELISA Histology	1.54 1.95	1.30–1.83 1.26–3.01	9.14% 42.20%	0.36 0.19	0.97	0.33
	**Age** < 60 years ≥ 60 years	1.53 1.63	1.19–1.98 1.33–2.00	0.00% 21.95%	0.45 0.23	0.14	0.71
**Case-Control**	**Continents** Africa Asia Europe	2.19 2.82 3.71	1.02–4.69 2.24–3.55 2.07–6.64	91.17% 73.09% 55.82%	0.00 0.00 0.08	1.24	0.54
	**Type of Glaucoma** PACG PEG POAG	1.73 0.59 3.71	1.04–2.90 0.27–1.25 2.93–4.70	0.00% 85.29% 63.98%	0.72 0.01 0.00	25.08	0.00
	**Diagnostic Method of HP** DIGFA ELISA Histology UBT	2.58 2.55 4.06 3.01	1.20–5.52 1.87–3.46 2.28–7.22 2.12–4.28	71.34% 83.76% 37.21% 43.16%	0.06 0.00 0.19 0.10	2.14	0.54
	**Age** < 60 years ≥ 60 years	2.75 2.95	1.97–3.83 2.27–3.83	66.19% 75.69%	0.00 0.00	0.11	0.74

## Discussion

In recent decades, HP infection has been considered a critical risk factor for glaucoma. However, the study sample size is limited, and the studies have conflicting results. Therefore, we conducted this meta-analysis to determine the relationship between Helicobacter pylori and primary open-angle glaucoma.

In the present meta-analysis, twenty-four studies were reviewed and included. These studies included case-control and cohort studies, and each study was separately analyzed. The results of this meta-analysis, both in case-control studies and cohort studies, showed the relationship between HP and Glaucoma. In addition to the overall association of Glaucoma with HP in this study, subgroup analysis showed the association of HP infection with POAG, NTG, and PXFG. In addition, subgroup analysis based on geographical location in the present meta-analysis showed that glaucoma patients are at higher risk of HP infection if they live in European countries. Also, in this meta-analysis, the age of Glaucoma patients in selected primary studies was divided into groups less than or equal to 60 years and more than 60 years. The analysis showed that glaucoma over 60 increases the risk of HP infection.

Glaucoma is the second leading cause of blindness worldwide after cataracts [10] and the leading cause of irreversible blindness, but many aspects of its pathogenesis remain unknown. Several possible mechanisms could support the fact that HP infection increases the risk of glaucoma. One is that HP infection may influence the pathophysiology of glaucoma by releasing various proinflammatory and vasoactive substances and affecting the apoptotic process [50]. HP infection is responsible for inflammation, increased production of reactive oxygen species, and induction of oxidative DNA damage in the gastric mucosa [51]. HP infection locally induces a chronic inflammatory state consisting of neutrophils, polymorphonuclear cells (PMN), and lymphocyte recruitment at the site of infection. The PMN cells attempt to damage bacterial structures by producing superoxide radicals and other ROS (Reactive Oxygen Species), thus determining local oxidative stress. And finally, excessive and long-term production of ROS in the gastric mucosa may damage cellular components such as unsaturated fatty acids, proteins, and DNA, and lipid peroxidation of the membrane will lead to disruption of various cell membrane and organelle functions [52]. Ascorbic acid is considered a primary substrate in eye protection due to its high ocular concentration [53]. This molecule is abundant in the aqueous humor, cornea, and tear film. Vitamin C is an important antioxidant that protects cells from death caused by oxidative stress. Probably, HP can damage the eyes and cause Glaucoma in this way. Therefore, HP can release various inflammatory factors such as cytokines, C-reactive protein, and nitric oxide [54]. Another mechanism is that HP infection can stimulate platelet and platelet-leukocyte aggregation, leading to decreased ocular blood flow and ocular ischemia [28].

Another point is that studies have proven that glaucoma patients have a common genetic factor that makes them more susceptible to HP infection [32]. In addition, the toxic substances secreted by HP may affect glaucoma and cause antibody-induced apoptosis that leads to inflammation in the retrobulbar region [18]. But the most likely mechanism of connection is an autoimmune reaction. In this way, the anti-HP antibody may cross the blood-aqueous humor barrier, condense in the aqueous humor and cause or aggravate glaucomatous damage [24].

As mentioned, there are various studies on the relationship between HP and Glaucoma and its types, including POAG. For example, some studies have a confirming role in this connection and investigate the condition of Glaucoma with the treatment of Helicobacter pylori. In a study by Shahram Ala et al., it was stated that HP eradication treatment might positively affect glaucoma management. The study results showed that intraocular pressure decreased significantly after HP eradication treatment in the intervention group. At the same time, this was not the case in the control group that was not treated with HP [26]. The same result was obtained in another study by Kountouras with a smaller amount [23].

Other studies have documented a higher prevalence of HP in POAG. These studies concluded that HP infection is much more common in patients with primary open-angle glaucoma [19, 40]. In another study conducted by Tsolaki et al., a positive correlation was found between HP infection and dementia, HP infection and glaucoma, and also between dementia and glaucoma [6]. However, some studies reported conflicting results. For example, one study reported that HP infection and seropositivity for Cag-A virus strains carrying HP were not significantly associated with any type of glaucoma [39]. In this context, a meta-analysis study has also been conducted, which, similar to the results of the present study, showed a statistically significant relationship between HP infection and Glaucoma, and further analysis showed that this positive relationship is only observed in POAG and NTG patients, which were consistent with the results of the study. But the mentioned study did not find an association between HP and PXFG [20]. But in the present study, there was also a relationship between HP and PXFG. Another meta-analysis study conducted in 2020 by Doulberis et al. examined the relationship between active HP infection and glaucoma. The results of this study also showed that the overall relationship between HP infection and Glaucoma was statistically significant. However, the degree of heterogeneity between the results of the studies was high. Also, in Doulberis et al. study, there was only a relationship between HP, POAG, and NTG but not PEG. Regarding the effect of geographic location, similar to our research, it was found that people living in European countries with glaucoma are more exposed to HP infection [31].

The current study had strong points compared to previous meta-analysis studies, including that the last field meta-analysis [31] was conducted in 2020 and on seventeen studies. After that, other case-control and cohort studies investigated the relationship between HP and glaucoma, which were added to the present meta-analysis. There was no heterogeneity in the results obtained in pooled cohort studies, which is a strong point compared to the previous investigation. The heterogeneity after combining the case-control studies was higher in pooled case-control analyses than in the cohort studies. This can be attributed to selecting cases and controls from a common source or using tools with different methods to identify the desired outcome. Also, in addition to OR, meta-analysis RR was calculated.

In addition to these strengths, the Egger test results showed an evident and significant publication bias in the combination of studies included in our analysis. However, the cutting and filling method did not show publication bias. Nevertheless, despite a comprehensive search of all relevant articles, publication bias is still unavoidable.

The presence of our important confounding variables between glaucoma and Helicobacter pylori may help health policymakers, and clinical professionals make decisions. One of the limitations of this meta-analysis is the lack of subgroup analysis based on confounding variables based on the presence of chronic diseases such as diabetes, HTN, and … or parameters like BMI. This is the need for more reporting of the desired relationship in the present meta-analysis based on these variables in selected primary studies. Future studies should be conducted considering these variables with a large sample size.

## Conclusions

The present study provided strong evidence regarding the relationship between HP and Glaucoma. Based on the results of this study, HP is considered a risk factor for Glaucoma, as well as its types, including POAG, NTG, and PXFG. Many factors can cause Glaucoma, and simply by eradicating Helicobacter pylori, we can’t expect that glaucoma will disappear; only perhaps its incidence will decrease. So, further studies and sufficient evidence on a larger scale are needed to show that the elimination of Helicobacter pylori infection positively impacts glaucoma parameters.

## Data Availability

The datasets used and analyzed during the current study are available from the corresponding author upon reasonable request.

## Abbreviations

CI: Confidence Interval
ELISA: Enzyme-Linked Immunoassay
EMBASE: Excerpta Medica Database
HP: H. Pylori, Helicobacter Pylori
HTN: Hypertension
IOP: Intraocular Pressure
NOS: Newcastle Ottawa Scale
NTG: Normal-Tension Glaucoma
OR: Odd’s Ratio
PACG: Primary Angle-Closure Glaucoma
PMN: Polymorphonuclear cells
POAG: Primary Open-Angle Glaucoma
PRISMA: Preferred Reporting Items for Systematic Reviews and Meta-Analyses
PXFG: Pseudo-Exfoliation Glaucoma
RCG: Retinal Ganglion Cell
RCT: Randomized controlled trial
ROS: Reactive Oxygen Species
RR: Risk Ratio
SACG: Secondary Angle-Closure Glaucoma
SD: Standard Deviation
SOAG: Secondary Open-Angle Glaucoma
UBT: Urea Breath Test
